# Acute High Intensity Interval Exercise Promotes Circulating Progenitor Cell Mobilization and Improves Microcirculation in Patients with Chronic Heart Failure

**DOI:** 10.3390/jcdd13060262

**Published:** 2026-06-11

**Authors:** Georgios Mitsiou, Savvas P. Tokmakidis, Irini Patsaki, Katherina Psarra, Christos Kourek, Eleftherios Karatzanos, George Papathanasiou, Stavros Dimopoulos

**Affiliations:** 1 Cardiopulmonary Exercise Testing and Rehabilitation Laboratory, 1st Critical Care Medicine Department, Evangelismos Hospital, National and Kapodistrian University of Athens, 10676 Athens, Greece; gmitsiou@uniwa.gr (G.M.); chris.kourek.92@gmail.com (C.K.); lkaratzanos@gmail.com (E.K.); 2 Department of Physiotherapy, University of West Attica, 12243 Athens, Greece; ipatsaki@uniwa.gr (I.P.); gpapa@uniwa.gr (G.P.); 3 Clinical Ergophysiology and Exercise Physiology Laboratory, School of Physical Education and Sport Science, Democritus University of Thrace, 69100 Komotini, Greece; stokmaki@phyed.duth.gr; 4 Immunology and Histocompatibility Department, Evangelismos General Hospital, 10676 Athens, Greece; kpsarra@outlook.com; 5 Cardiac Surgery Intensive Care Unit, Onassis Hospital, 17674 Athens, Greece

**Keywords:** endothelial progenitor cells, hematopoietic progenitor cells, interval exercise, microcirculation, vascular endothelial growth factor

## Abstract

Endothelial progenitor cells (EPCs) constitute a cell population that enters the circulation during aerobic exercise and facilitates vascular function. In a similar action, hematopoietic progenitor cells (HPCs) are also released into circulation in response to exercise. Peripheral vascular dysfunction is frequently present in patients with heart failure. Whether acute interval exercise performed with high intensity induces EPC and HPC mobilization and affects microcirculation remains under investigation. The study population consisted of nineteen male patients with chronic heart failure (CHF) and eleven age-matched healthy individuals who underwent a high-intensity interval exercise session. Blood was drawn before, immediately after exercise, and 40 min after exercise to identify the numbers of circulating EPCs and HPCs by flow cytometry. Microcirculatory assessment was performed using near-infrared spectroscopy before and after exercise. Vascular endothelial growth factor (VEGF) change was also assessed before and after exercise in patients with CHF using flow cytometry. The interval exercise protocol revealed significant effects (*p* < 0.05) on EPC and HPC mobilization and systemic microcirculation (*p* < 0.05) in patients with CHF and healthy individuals. No significant differences were observed between patients with CHF and healthy individuals during interval exercise. VEGF did not reveal any changes immediately after interval exercise in CHF patients. Acute high-intensity interval training was associated with increased EPC and HPC mobilization and changes in microcirculation in patients with CHF and healthy individuals.

## 1. Introduction

Circulating progenitor cells modulate the immune response, preventing the progression of vascular damage and reducing inflammation and cardiovascular risk [1]. An increased number and function of progenitor cells enhance vascular integrity and promote angiogenesis [2]. Endothelial progenitor cells (EPCs) constitute a type of bone marrow-derived progenitor cells that promote the normal function of the endothelium to maintain adequate vascular homeostasis, produce protective molecules, and prevent the development of vascular disease [3]. Hematopoietic progenitor cells (HPCs) constitute a related cell population with progenitor-like properties [4]. These are another type of bone marrow cells, distinct from EPCs, with a capacity to develop and maintain all blood-forming tissues, regulated in patients with endothelial dysfunction. Moreover, HPCs have a role in regeneration as potential indicators to improve reparatory mechanisms in the vascular system [4].

The vascular system in patients with heart failure, endothelial function, and mobilization of endothelial cells could also be affected by regular exercise [5]. The effect of exercise on vasculature is closely related to the synthesis of nitric oxide mediated through intermittent increases in endothelial shear stress and vascular endothelial growth factor (VEGF) release [6]. During exercise, EPCs circulate into the bloodstream, ‘home in’ on sites of ischemic vascular injury, facilitate endothelial function, and influence vascular regeneration [7,8]. In this process, there is a coordinated interaction between EPCs and HPCs, which can also circulate into the bloodstream after exercise and participate in new vessel formation by vasculogenesis [9]. In patients with heart failure, however, the training intensity requires further attention and has not yet been clarified.

Studies indicate that exercise enhances endothelium-dependent vasodilation in forearm circulation in healthy individuals [10], hypertensive subjects [11], and patients with chronic heart failure (CHF) [12]. Taking into consideration that CHF is associated with peripheral endothelial dysfunction, the beneficial impact of exercise on microcirculation has been the subject of research. Continuous exercise has been shown to improve peripheral microcirculation, restore vasomotor function, and maintain endothelial integrity [13], but high-intensity interval exercise needs to be further clarified.

Although continuous exercise of moderate intensity has been the standard form of exercise to promote cardiorespiratory endurance, some studies have shown that interval exercise training of high intensity can also improve cardio-respiratory fitness [14], promote endothelium integrity [15], and reverse the risk factors of cardiovascular disease [16]. Continuous training appears to be an effective physiological stimulus to liberate EPCs and angiogenic cells into the circulation [17]. However, the impact of acute interval exercise on the mobilization of EPCs and HPCs has not been thoroughly identified. Furthermore, limited data indicate that in healthy individuals and in patients with cardiovascular disease, the effects of interval exercise training on EPC mobilization appear to be controversial [18,19,20,21] and require further verification. Moreover, there is also a lack of data concerning patients with heart failure. Thus, it is valuable to examine if the exercise stimulus in an interval form can induce the mobilization of EPCs and HPCs or improve endothelial reactivity and increase VEGF concentration in patients with CHF. The purpose of the present study was to investigate the impact of a high-intensity interval exercise protocol on the mobilization of EPCs and HPCs, micro-vascular reactivity, and changes in VEGF serum levels.

## 2. Materials and Methods

### 2.1. Study Population

The patients were referred to our laboratory from the Heart Failure Clinic of our institution to undertake a Cardiopulmonary Exercise Test (CPET) as a requirement to participate in the study. Patients were on a stable optimal medical regimen for at least 3 months. If they were clinically stable, without hospital admission in the previous 3 months, and without participation in any other exercise program in the previous 6 months, they were eligible to participate in the study. The criteria of exclusion from the study consisted of contraindications for the CPET (according to the American Thoracic Society/American College of Chest Physicians Statement on CPET) [22], unstable angina or recent acute myocardial infarction, and conditions such as cancer and liver or kidney disease in which neo-vascularization might have been present. Additional exclusion criteria were uncontrolled hypertension, serious vascular disease, serious chronic obstructive pulmonary disease, peripheral arterial disease, and neuromuscular disease. The qualified participants had no symptoms associated with active ischemia. A healthy matched control group was also included in the study. In accordance with the Helsinki declaration for human studies [23], all participants were informed about the risks and the benefits of the study and signed a consent form. Baseline data and clinical characteristics of all patients are presented in Table 1.

### 2.2. Study Design

This study was designed as an exploratory analysis aiming to examine the acute effects of interval exercise on progenitor cells, microcirculation, and VEGF in patients with CHF vs. healthy participants. All participants were familiarized with a cycling exercise and underwent a CPET. Maximal exercise testing was performed on a calibrated electronically braked bicycle ergometer in an upright position as previously described [24]. Patients and healthy individuals were verbally encouraged to exercise to exhaustion, usually defined as intolerable leg fatigue or dyspnea. Work rate increments were estimated using the Hansen et al. equation [24] to attain a test duration of 8 to 12 min. Gas exchange was measured breath by breath with the online system and was assessed while the subject was breathing through a low-resistance valve with a nose clamp, using a metabolic cart (Vmax 229; Sensormedics, Yorba Linda, CA, USA). Heart rate and rhythm were monitored by a 12-lead electrocardiography system (MAX1, Model 2000; Marquette Electronics, Milwaukee, WI, USA), arterial blood pressure was measured every 2 min, and oxygen uptake at peak exercise (VO_2peak_) was obtained by an average measurement during the last 20 s of the CPET. Peak work rate was defined as the highest work level reached and maintained at a pedaling frequency of no less than 60 rpm for 20 s. The results from the CPET were analyzed and used to determine the appropriate workload and intensity of training for each participant.

### 2.3. Exercise Protocol

The exercise load based on the CPET was individually prescribed to every participant. Baseline parameters of hemodynamic stability were recorded three minutes before exercise. The exercise training protocol started with a 5 min unloading-pedaling warm-up. The interval protocol initiated with 3 min cycling at 50%VO_2peak_. Then, each participant performed four cycling intervals of 4 min duration at an intensity of 80%VO_2peak_. Each interval was interspersed by a 3 min active recovery at 50%VO_2peak_ (VO_2max_ was prescribed for healthy participants, respectively). The training session ended after the last 3 min of active recovery, which was used as a cool-down phase (31 min total duration; 16 min work and 15 min active recovery).

**Figure 1:**
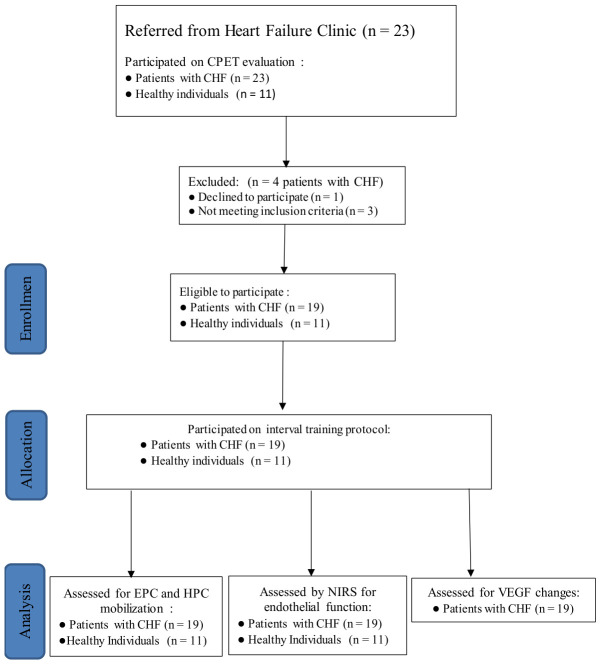
Flow chart of the study. EPCs: endothelial progenitor cells; HPCs: hematopoietic progenitor cells; NIRS: near-infrared spectroscopy; VEGF: vascular endothelial growth factor.

In addition, all participants were evaluated by near-infrared spectroscopy (NIRS) 3 min before and 3 min after the exercise training protocol. VEGF serum levels were also assessed in patients with CHF. The flow chart of the study is summarized in Figure 1.

### 2.4. Measurements

#### 2.4.1. Blood Samples and Flow Cytometry

Fasting venous blood samples were drawn from an antecubital vein with the use of a catheter 2 min before exercise, immediately after exercise, and 40 min after exercise. Blood was collected in 8.6 mL acid-citrate-dextrose tubes at 4 °C temperature, and whole blood samples were analyzed within 2–3 h using flow cytometry for a complete EPC and HPC blood count analysis [25]. During sample processing/preparation, the blood was centrifuged twice at 700 rpm (i.e., 55× *g*) for 20 min without a brake in the blood centrifuge. After the first centrifugation, the upper phase of the blood plasma was removed and stored in a separate 0.25 mL tube. The lower phase of the plasma, which contained the red blood cells, was resuspended using 10 mL of cold 1× PBS (Phosphate-Buffered Saline: used in chemistry to separate and dissolve biological samples) containing 0.5% BSA (Bovine Serum Albumin: used as a compensating agent to reduce nonspecific interactions between antibodies and antigens during the immunoassay detection process) and 1.5 m EDTA (Ethylenediaminetetraacetic acid: used as an anticoagulant to maintain the integrity and reduce the degradation of biological samples during centrifugation) to be followed by the second centrifugation (700 rpm or 55× *g* for 20 min). Following the second centrifugation, 2.5 mL was transferred into a separate tube and kept on ice. Concurrently, 500 μL of the samples were also transferred into sample tubes and four antibodies were added (see Table 2). Then, 9 mL of ACK lysing buffer was added (to lyse red blood cells), before proceeding to the third centrifugation at 250 rpm (i.e., 7× *g*) at 4 °C, gradually braking at 5 min and incubation of the sample at room temperature (18 to 25 °C) for 3 min. Finally, the sample preparation process was completed by washing the cells again twice with 10 mL of cold 1× PBS, and two more centrifugations were performed under the previous conditions. All samples were placed in special tubes and transferred for analysis to the flow cytometer up to 2.5 h after blood was drawn. An acquisition gate was established that included mononuclear cells but excluded most granulocytes and debris; 10^6^ mononuclear events were routinely collected to visualize and gate on this population.

**Figure 2:**
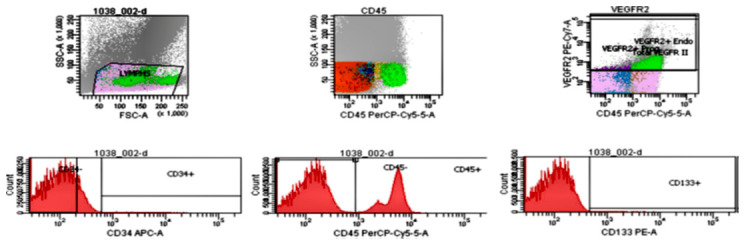
Representative dot plot analysis for endothelial progenitor cell (EPCs) and hematopoietic progenitor cell (HPCs) determination with the Boolean analysis. Cells were gated based on forward and side scatter to exclude debris, followed by doublet discrimination. In all samples, CD133 is expressed positively for EPC enumeration and negatively for HPC enumeration. The negative and positive by definition cell populations for each particular monoclonal antibody were used as internal negative and positive controls.

Flow cytometry was performed in the Immunology–Histocompatibility Department Flow Cytometry Laboratory using BDFACSCantoII (Becton Dickinson fluorescence-activated cell sorting, Becton, Dickinson and Company, San Jose, CA, USA). The Boolean technique was applied for result analysis using combinations of specific surface markers (Figure 2). To define EPCs, two combinations of antigens were applied. The measured population number of EPCs was based on the surface expression of four antigens: CD34/CD45/CD133 (cluster of differentiation) and KDR (kinase domain receptor). The latter is the extracellular domain of vascular endothelial growth factor receptor-2 (VEGFR_2_^+^). Depending on the expression of the endothelial antigen, the CD34^+^/CD133^+^/KDR and CD34^+^/CD45^−^/KDR cell populations were considered EPCs_1_ and EPCs_2,_ respectively. For the HPC evaluation, the markers CD34^+^/CD45^−^/CD133^−^ defined HPCs. The entire blind preparation procedure was conducted by an expert in flow cytometry.

#### 2.4.2. Assessment of Endothelial Function

In order to assess the effects of exercise on vascular microcirculation, the NIRS apparatus was used [26]. The NIRS’s probe was placed over the thenar eminence [27] and a 3 min branchial occlusion on the vascular system was performed by inflating a pneumatic cuff 50 mmHg above the systolic pressure. The main micro-circulation parameters were: (i) tissue oxygen saturation (StO_2_%); (ii) oxygen consumption rate (%/min) as calculated by the regression line of the first minute of StO_2_ decay after occlusion; (iii) oxygen reperfusion rate, representing endothelial function (%/s), by quantifying the thenar hyperemic reactive response occurring after the release of vascular occlusion; and (iv) reactive hyperemia, expressed as the percentage of the signal change during arterial occlusion and estimated by vascular reserve (%/s). Endothelial reactivity was observed according to StO_2_ signals and their changing velocity until they gradually returned to baseline and perfusion was re-established. All NIRS measurements were stored and analyzed offline using the proprietary software Hutchinson InSpectra Analysis (Hutchinson Technology Inc., Hutchinson, MN, USA version 2.4).

#### 2.4.3. VEGF Assessment

The measurement of VEGF serum levels was performed using the Human Soluble Protein Flex Set System, which includes a set of antibodies designed to detect VEGF in peripheral blood. The Cytometric Bead Array analysis was also adopted for capturing a soluble analyte or a set of analytes using beads of known size and fluorescence, enabling the detection of sandwich complexes (capture bead + analyte + detection reagent) using flow cytometry. During the process, the upper phase (plasma) of venous blood after centrifugation was used for measuring VEGF serum levels. Subsequently, antibodies selectively targeting VEGF were bound to a solid substrate. Finally, the plasma sample was incubated with these antibodies, and the concentration of VEGF was isolated and recorded. The quantity of VEGF in the plasma was measured based on the sample’s reaction to a series of standard VEGF gradients, used to generate a dose–response curve. Four-color flow cytometry was performed using a Navios flow cytometer (Beckman Coulter, Beckman Coulter, Inc., Brea, CA, USA). Values were expressed as standard deviation (SD) pg/mL.

### 2.5. Statistical Analysis

Data were expressed as mean ± SD. Normality of distribution was assessed with the Shapiro–Wilk test. Differences at the three-time points of measurement on EPC and HPC mobilization were assessed with factorial analysis of variance (ANOVA) 1 × 3 (protocol × time) with repeated measures. Differences at the two time points of measurement on microcirculation and VEGF were assessed using the ANOVA 1 × 2 (protocol × time) with repeated measures. A two-way repeated measures ANOVA (group × time) was also performed as an exploratory post hoc analysis to examine the interaction between group and time. The Mann–Whitney U test was adopted to compare age distributions between groups. The Sidak correction was applied for post hoc multiple comparisons. The effect size was calculated with Cohen’s d as the mean of the difference/SD of the difference to provide low (small ≥ 0.2), medium (moderate ≥ 0.5), and high (large ≥ 0.8) difference effects between two variables. Statistical significance was accepted at *p* < 0.05 and all computations were accomplished by the SPSS statistical package version 28 (SPSS Inc., Chicago, IL, USA).

## 3. Results

Twenty-three heart failure patients were requested to participate in the study. One declined for personal reasons and three failed to meet the inclusion criteria. Nineteen non-cachectic male outpatients with stable CHF were eligible to participate and eleven healthy individuals started the experimental process and completed the study. No significant difference in age was observed between groups (*p* = 0.2). A significant group × time interaction was also not observed (*p* = 0.3). The flow chart of the study is illustrated in Figure 1.

### 3.1. Mobilization of EPCs and HPCs

**Figure 3:**
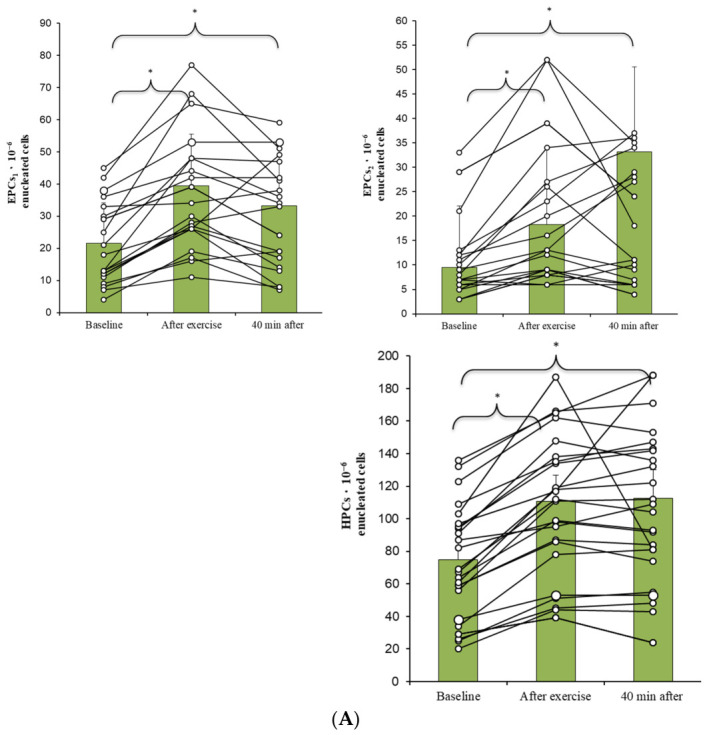

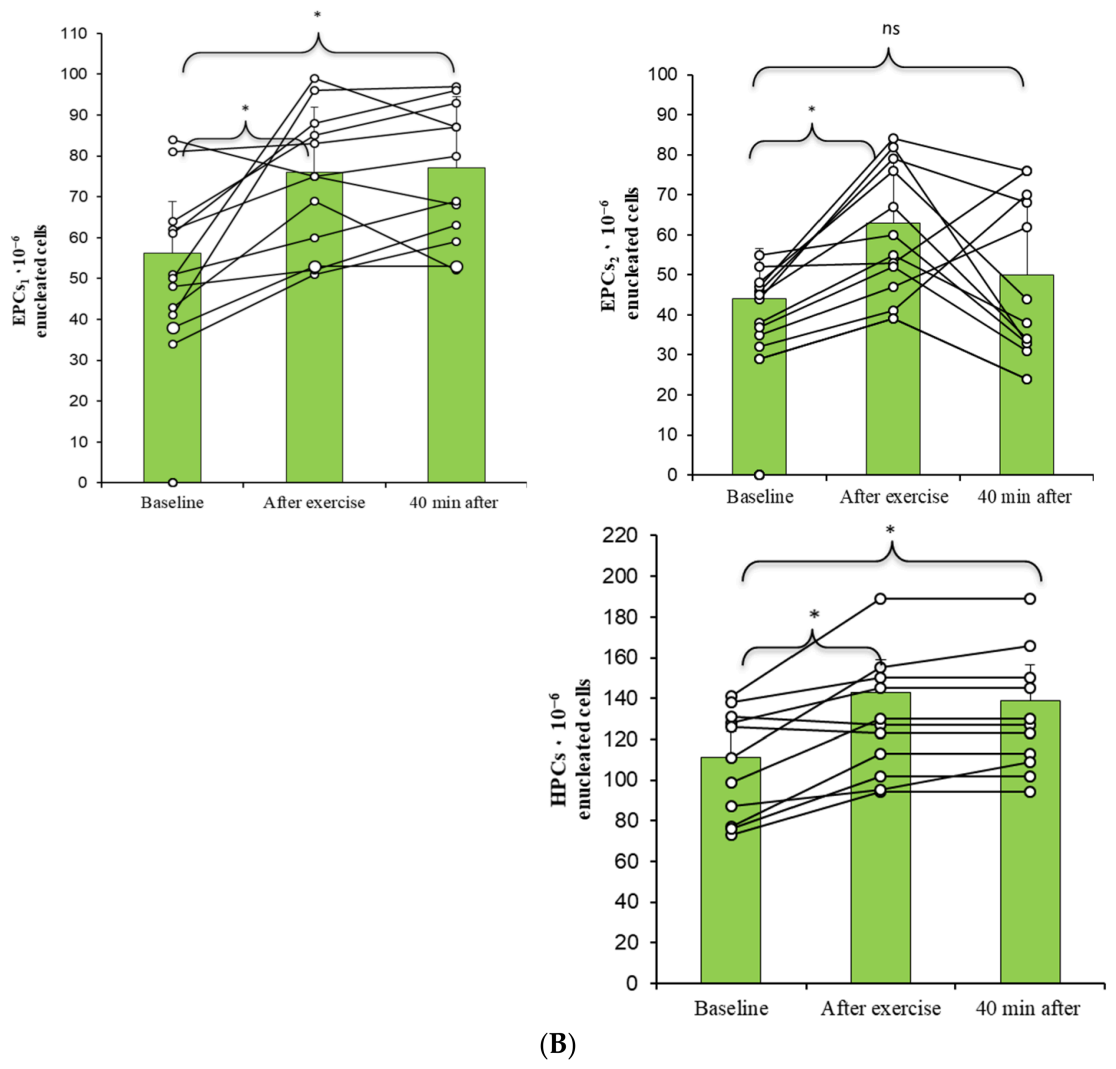
Individual values of endothelial progenitor cells (EPCs) and hematopoietic progenitor cells (HPCs), identified by a combination of different markers, depicting the interval training before, after, and 40 min after in patients with chronic heart failure (*n* = 19) (**A**) and healthy individuals (*n* = 11) (**B**). Error bars reflect standard deviation, * *p* < 0.05 vs. baseline; ns: non-significance.

Based on the combined identification markers of progenitor cells, interval exercise revealed significant time main effects on EPCs_1_, EPCs_2_, and HPCs immediately after exercise (EPCs_1_: *p* = 0.001; EPCs_2_: *p* = 0.001; HPCs: *p* = 0.01) and at 40 min after exercise (EPCs_1_: *p* = 0.04; EPCs_2_: *p* = 0.05; HPCs: *p* = 0.01) in patients with CHF. Furthermore, the statistical analysis revealed significant effect on EPCs_1_ and HPCs immediately after exercise (EPCs_1_: *p* = 0.001; EPCs_2_: *p* = 0.001; HPCs: *p* = 0.03) and at 40 min after exercise (EPCs_1_: *p* = 0.01; HPCs:, *p* = 0.01), except for EPCs_2_ at 40 min (*p* = 0.06) after exercise (Figure 3) in healthy individuals (Table 3). No significance was observed between the two groups immediately (*p* = 0.06) and 40 min after exercise (*p* = 0.08).

### 3.2. Systemic Microcirculation

In both groups of patients and healthy individuals, NIRS measurements at the thenar muscle, representing the systemic circulation, revealed that the oxygen consumption rate increased significantly after exercise (main effect in CHF, *p* = 0.01; main effect in healthy individuals, *p* = 0.04). The reperfusion rate following the release of vascular occlusion increased (*p* = 0.001). The time to baseline significantly declined after exercise (*p* = 0.05), and thus, reactive hyperemia was also affected in patients with CHF and healthy individuals (Table 4).

### 3.3. Vascular Endothelial Growth Factor Changes

No significant changes were revealed in VEGF serum levels before and after exercise in patients with CHF (*p* > 0.05).

## 4. Discussion

The main findings of the present study indicate that an exercise training session in an interval mode of higher intensity can provoke EPC and HPC mobilization and changes in microcirculation in CHF and healthy subjects, facilitating vascular function.

### 4.1. Effects of the Training Protocol

The exercise protocol used in the present study was found to be effective in mobilizing EPCs and HPCs and providing benefits to vascular function. An increase in EPC levels was associated with a concurrent effect on endothelium reactivity. Data regarding the effects of exercise intensity on EPC mobilization in patients with cardiovascular disease are limited and controversial [20,21,28]. Furthermore, the latter studies were not conducted in acute settings. The total work output of exercise during a training session appears to be an important factor, with the intensity and duration also playing a significant role. In a previous study, we showed that during acute interval exercise in patients with CHF, the short duration may be counter-balanced by the high intensity of exercise stimulus and still be able to stimulate progenitor cell mobilization similar to long-duration exercise [29].

Some studies used short-term [7,30] or long-term [31] continuous exercise protocols, but only a limited number of studies [20,21] were conducted with high-intensity interval exercise training to evaluate the mobilization of EPCs and were able to reveal debatable results. High intensity at interval exercise (80% VO_2peak_/VO_2max_) provides a greater peripheral stimulus with beneficial effects on CHF patients at the endothelial and tissue levels when using a shorter period of time [14]. Furthermore, increasing exercise intensity is efficient to increase cellular and molecular responses due to changes in shear stress on the walls of blood vessels as compared to low-intensity exercise [32,33]. In our study, the higher intensity during the interval work phase with a demanding workload was effective in enhancing EPC and HPC mobilization.

### 4.2. Exercise-Induced Endothelial Cell Mobilization

Laufs and colleagues [34] performed three different exercise protocols in healthy volunteers and demonstrated that intensive and moderate exercise of 30 min (but not 10 min with moderate intensity) increased circulating levels of EPCs. Continuous exercise as a stimulus for EPC mobilization has also been proven to be effective in different groups of patients with heart disease, including coronary artery disease [35] and heart failure [36]. More specifically, exercise protocols with a certain duration (20 min) of continuous exercise [8,31] promote EPC mobilization in CHF patients, followed by improved vascular function. Likewise, the exercise duration attained in the present study agrees with other studies [14,34] and indicates that at least 20 to 30 min of exercise is needed to exert beneficial effects on cardiovascular health. However, the impact of interval exercise in patients with cardiovascular disease has not yet been established. In healthy subjects, 3 min intervals at 40 and 80% VO_2max_ [19] have been found to enhance EPC mobilization by augmenting the activity of stromal cell-derived factor-1 (SDF-1a) and the matrix metalloproteinase-9, vascular endothelial growth factor-A (VEGF-A), and nitric oxide bioavailability. In another study, however, 60 s of exercise consisting of 10 s intervals at 120% of the pre-training peak work rate [18] appears to have no impact on EPC mobilization. High-intensity interval training in heart failure patients used by Wisløff et al. [14] yielded improvements in endothelial function, through adjusting the intensity of exercise training to peak heart rate. In our study, the interval protocol (4 × 4 min at 80% of VO_2peak_/VO_2max_) corresponding to an intensity over 90% peak heart rate) was selected as an established protocol [14]. Thus, we showed that the stimulus of exercise with higher intensity improved mobilization of EPCs and HPCs in patients with CHF and healthy individuals. Taking into consideration the main effects of our study, an increased mobilization capacity was observed when different combinations of markers were used to define progenitor cells (EPCs and HPCs) immediately after exercise, as well as 40 min after the interval exercise session. Indeed, training intensity induces greater adaptations in the cardiovascular system, aerobic capacity, energy reserves, and endothelial function [14]. However, more evidence is required on the dose–response. Finally, the immediate post-exercise increase in circulating progenitor cells may reflect not only bone marrow mobilization but also transient redistribution from peripheral compartments, which has been described in response to tissue hypoxia. However, the use of specific antigens (particularly CD34^+^) shows progenitor cell mobilization rather than an increase in circulating mature stem cells, which are more likely to reflect redistribution. Notably, the exercise-induced mobilization of EPCs and HPCs was even better immediately after exercise than 40 min after exercise, depicting that the observed increase may better reflect true mobilization. This is further supported by previous studies that observed increased EPC numbers immediately after or within the first minutes following acute maximal exercise in patients with cardiovascular disease and healthy individuals [37,38].

### 4.3. Kinetics of EPCs and HPCs After Exercise

Although several studies have established the role of EPCs in vascular formation, only a few studies have examined the endogenous kinetics of these cells. Assessment of EPC kinetics showed that EPCs increase after exercise and remain high even 48 h after the intervention [30]. In our study, we observed an elevation in progenitor cells immediately after exercise. And this elevation remained almost unchanged up to 40 min after exercise, thus affecting the endothelium function. Possible triggers of this elevation are shear stress and the activation of cytokines and chemokines such as SDF-1a, which are important regulators of progenitor cell activation and trafficking. In addition, there is evidence that interval exercise improves endothelium-dependent vasodilation and promotes the release of nitric oxide, which activates VEGF-A [38]. Furthermore, the interval protocol was also effective in elevating HPCs immediately after exercise, as well as 40 min after the completion of exercise. Our findings clearly demonstrate that the chosen stimulation of exercise was adequate to induce the appropriate cellular responses. Subsequently, EPC and HPC kinetics may be regarded as a physiological counteraction to maintain an intact endothelial cell layer and contribute to vascular development. Moreover, in contrast to therapeutic approaches, which require selective cell infusions in the body, our study provides evidence that high-intensity interval exercise may prove to be a physiological stimulus to provoke cellular mobilization within the human body, avoiding the risk of any adverse events. However, a possible confounding effect of pharmacological therapy should be acknowledged since the eligible patients with CHF were receiving medications (b-blockers, ACE inhibitors, etc.; see Table 1) that might modulate the increase in circulating progenitor cell levels and endothelial function during exercise. Although some medications were used only by 53% (statins) and 31% (nitrates) of the eligible patients, the potential impact of pharmacological therapy on pro-angiogenic circulating progenitor mobilization cannot be excluded. These medicines indirectly support vascular homeostasis by attenuating inflammatory processes mainly via the modulation of oxidative stress pathways and improvement in NO bioavailability [39]. Their integration with exercise for the treatment of heart failure may offer synergistic effects, especially in patients with profound EPC impairment.

### 4.4. Markers of Endothelial Cell Identification

A controversy exists with respect to the true nature and origin of circulating EPCs since there are no unique or specific protein markers that can be used to determine a unique EPC population. The dominant endothelial cell population, named as endothelial colony-forming cells [40], was mainly defined upon culture isolation. In our study, however, the adoption of the EPC term was defined by flow cytometry. These cells are widely considered to represent a population of pro-angiogenic hematopoietic progenitors that support neovascularization. Various surface markers were combined to better characterize EPCs and increase the reliability of quantification. In some cases, the measurements were duplicated, and no differences were noticed after duplication. Initial studies regarding EPC detection suggest that CD34^+^ cells that express KDR may represent a phenotypically distinct population of putative EPCs that facilitate vascular function [7]. However, accumulating evidence suggests that putative EPCs have a hematopoietic origin and are likely to represent pro-angiogenic hematopoietic progenitors rather than definitively endothelial-committed cells [41]. It seems that the EPC definition now encompasses a variety of cells, including haemopoietic stem and progenitor cells. Indeed, EPCs are considered circulating pro-angiogenic progenitor populations that have a protective role in vascular homeostasis by forming functional blood vessels [42] or by local secretion of pro-angiogenic factors with paracrine effects on the cells forming vessels [43] rather than directly differentiating into mature endothelial cells themselves. Circulating CD34^+^ hematopoietic precursor cells are distinct from EPCs when VEGFR_2_^+^ is not expressed and may be the source of so-called outgrowth endothelial cells. Moreover, CD34^+^ was used for the purification of primitive hematopoietic progenitors [44]. Other antibodies, such as CD38 and CD90, which have been considered to be markers of hematopoietic precursor identification, were not included in our study. In addition, CD133^+^ cells are highly expressed on EPCs but disappear when differentiated, whereas CD133^−^ cells develop differential phenotypic equilibrium and display a differential gene profile, which is expressed through CD133^−^ cells on mature hematopoietic and endothelial cells [45].

### 4.5. Acute Microcirculatory Vascular Reaction

Vascular reactivity as measured by NIRS using the vascular occlusion technique has been shown to reflect the prognosis of patients with cardiovascular disease [46]. The participants in our study confirmed that exercise is an effective stimulus to improve microvascular oxygenation in a peripheral skeletal muscle bed not directly involved in muscular work at the active point of intervention. Overall, the interval protocol improved the oxygen reperfusion rate and microvascular oxygen kinetics.

### 4.6. Limitations of the Study

The relatively small sample size is a limitation in our study, as it may have been underpowered to detect modest between-group differences, reducing statistical power and limiting the generalizability of the findings. However, the sample was sufficient to demonstrate statistically significant results due to a high effect size. Power calculation was not performed because previous studies, although conducted in small cohorts, have not reported effect sizes for exercise-induced changes on EPC numbers in patients with CHF. Therefore, the present study should be considered exploratory, and the sample size may limit the robustness and generalizability of the findings.

Although we used several markers to identify circulatory progenitor cells, variations in measurements and extreme individual responsiveness might not be avoided. Moreover, the group of patients consisted exclusively of male participants. Investigation into the circulating progenitor cell response during interval exercise in the female population should be further warranted. Biological sex is known to influence circulating progenitor cell biology [47], vascular function, and responses to exercise interventions. These limitations compromise the generalizability of the findings to females. In addition, the impact of a single exercise session or even a maximal exercise test on EPC mobilization and microcirculation in patients with CHF and healthy individuals is conflicting, and this study clarifies the immediate effects of exercise on progenitor cell mobilization. However, the present study is limited to the effects of a single exercise session and therefore cannot provide evidence for long-term benefits.

Another limitation is that the EPC definition encompasses a variety of cells, including haemopoietic stem and progenitor cells. Indeed, there is data supporting that CD34^+^/CD133^+^ define the hematopoietic progenitor cells [48]. Therefore, we have to point out that the data on CD34^+^CD133^+^ cell definitions are conflicting, and further phenotyping is necessary to discriminate EPCs from HPCs. Some technical considerations should also be considered. These include rare event flow cytometric analysis and platelets (if bound to cells) interference, elevated background signal, and difficulties that may occur in discriminating true events from artifacts at very low frequencies.

Finally, VEGF serum levels were examined as a secondary outcome, but were not found to increase immediately after exercise in patients with CHF. This might be attributed to the time point we chose to assess VEGF (immediately after exercise), since other studies suggest that a period of 15 to 20 min is predisposed to activate VEGF changes during acute exercise [38]. The non-significant response to VEGF changes during interval exercise may be attributable to the selected sampling time window, chosen to affect VEGF changes. The absence of intermediate sampling points indicates that transient alterations in VEGF levels cannot be excluded during interval exercise.

## 5. Conclusions

The present study indicates that acute high-intensity interval exercise induces EPC and HPC mobilization, increases vascular reactivity, and promotes microvascular oxygenation in male patients with CHF and healthy individuals. The exercise-induced mobilization of pro-angiogenic hematopoietic progenitor cells following an interval training session seems to be a safe and feasible physiological procedure that facilitates vascular function and positively affects microcirculation. Notably, cardiovascular medicines affect circulating progenitor cell properties. Therefore, the observed alterations may reflect, at least in part, the influence of pharmacological treatment. Nonetheless, further studies are required to assess the dose–response of exercise intensity and duration and reveal the underlying characteristics of exercise-induced health benefits in patients with CHF. Female participants should also be included, and potential sex-specific responses to exercise in patients with CHF need to be evaluated.

## Figures and Tables

**Table 1:** Descriptive and clinical characteristics of patients with CHF and healthy individuals.

Anthropometry	Patients with CHF (*n* = 19)	Healthy Individuals (*n* = 11)
Age (years)	49.2 ± 10.7	46.3 ± 8.6
BMI (kg/m^2^)	27.2 ± 3.1	27.7 ± 2.9
Body Mass (kg)	78.9 ± 18.6	82.9 ± 13.6
Body Height (cm)	175.4 ± 8.6	173.7 ± 8.6
Male/Female	19/0	7/4
VO_2peak_/VO_2max_ and LVEF		
VO_2peak_ (mL/kg/min)/VO_2max_ (mL/kg/min)	15.9 ± 2.7	31.8 ± 3.4
LVEF (%)	35.3 ± 3.5	63.2 ± 2.8
Heart Failure Characteristics No/%		
NYHA Class II	12 (63)	
NYHA Class III	7 (37)	
Non-ischemic	10 (53)	
Ischemic	9 (47)	
Arterial Hypertension	6 (31)	
Diabetes	5 (26)	
Dyslipidemia	4 (21)	
Smoking	―	
Medical Treatment		
B-blockers	16 (84)	
ACE inhibitors	15 (79)	
Nitrates	6 (31)	
Statins	10 (53)	
Anti-arrhythmics	11 (58)	
Anti-coagulants	6 (31)	
Anti-platelets	7 (37)	

CHF, chronic heart failure; LVEF, left ventricular ejection fraction; SD, standard deviation; ACE, angiotensin-converting enzyme; BMI, body mass index; NYHA, New York Heart Association. Continuous variables are expressed as mean ± standard deviation. Categorical variables are expressed as numbers and percentages.

**Table 2:** Characteristics of the antibodies used in flow cytometry.

Antibody	Source	Concentration	Catalog Number	Clone	Volume per Test
CD45-PerCP	BD Pharmingen	25 μg/mL	340665	2D1	10 μL
CD34-APC	BD Pharmingen	100 μg/mL	340441	8G12	3 μL
CD133-PE	Miltenyi Biotec	100 μg/mL	130-080-801	AC133	5 μL
VEGFR_2_ (KDR)-PE	R&D Systems	NA	FAB 3578	89106	5 μL

NA: not available.

**Table 3:** EPC and HPC mobilization after high-intensity interval exercise in CHF patients (*n* = 19) and healthy individuals (*n* = 11).

	Baseline	After Exercise		40 min After Exercise	
Participants	Cells × 10^−6^ Enucleated Cells	Cells × 10^−6^ Enucleated Cells	Effect Size/95%CI	Cells × 10^−6^ Enucleated Cells	Effect Size/95%CI
Endothelial Progenitor Cells (EPCs) identified by different markers EPCs_1_ markers: CD34^+^/CD45^−^/VEGFR_2_^+^					
CHF	21.5 ± 14.4	39.5 ± 24.4 ^a^	0.83 (−25.7 to −10.3)	33.2 ± 18.7 ^b^	0.77 (−18.9 to −5.1)
Healthy Individuals	56.2 ± 11.6	76 ± 15.1 ^a^	0.82 (−36.1 to −2.7)	77.5 ± 15.4 ^a^	0.70 (−36 to −6.1)
EPCs_2_ markers: CD34^+^/CD133^+^/VEGFR_2_^+^					
CHF	9.5 ± 7.2	18.3 ± 13.5 ^a^	0.73 (−14.1 to −3.7)	17.1 ± 14.5 ^b^	0.70 (−14.1 to −0.8)
Healthy Individuals	43.8 ± 9.3	63.2 ± 15.9 ^a^	0.82 (−31.3 to −7.3)	50 ± 19.0	0.70 (−25.4 to 9.8)
Hematopoietic Progenitor Cells (HPCs) HPCs markers: CD34^+^/CD45^−^/CD133^−^					
CHF	75.6 ± 35.0	110.6 ± 41.8 ^a^	0.75 (−44.2 to −28.8)	112.6 ± 38.7 ^a^	0.75 (−47.2 to −28.3)
Healthy Individuals	111.4 ± 62.6	143.7 ± 78.1 ^b^	0.59 (−64.2 to 12.4)	139.2 ± 66.7 ^a^	0.80 (−74.6 to 16.9)

CHF: chronic heart failure; EPCs: endothelial progenitor cells; HPCs: hematopoietic progenitor cells; VEGFR_2_^+^: vascular endothelial growth factor receptor-2. ^a^ *p* ≤ 0.01and ^b^ *p* ≤ 0.05 compared to baseline; all values are expressed as mean ± SD, standard deviation.

**Table 4:** Changes in microcirculatory parameters monitored by near-infrared spectroscopy and a vascular occlusion test, and in VEGF in patients with CHF (*n* = 19) and healthy individuals (*n* = 11).

	Baseline	After Exercise	95%CI	Effect Size
Tissue oxygenation (StO_2_)				
CHF	78.7 ± 3.7	79.0 ± 4.9	−2.4 to 0.87	0.25
Healthy Individuals	80.4 ± 4.4	79.5 ± 4.1	−2.2 to 0.85	0.30
StO_2_ downslope (%/min)				
CHF	10.1 ± 1.5	12.6 ± 1.9 ^b^	−2.8 to −0.07	0.80
Healthy Individuals	12.1 ± 2.4	14.9 ± 1.8 ^b^	−2.6 to −0.04	0.70
StO_2_ upslope (%/s)				
CHF	2.9± 0.7	3.7 ± 0.6 ^a^	−0.86 to −0.008	0.88
Healthy Individuals	3.2 ± 1.2	4.3 ± 1.1 ^b^	−0.74 to −0.1	0.69
Time to baseline (s)				
CHF	124.8 ± 37.3	111.7 ± 21.5 ^b^	−0.16 to 24.5	0.52
Healthy Individuals	109.3 ± 31.6	92.4 ± 26.9	−0.14 to 21.3	0.60
VEGF				
CHF	18.9 ± 14.9	21.2 ± 15.8	−1.24 to −0.6	0.27

CHF: chronic heart failure; StO_2_: tissue oxygen saturation; VEGF: vascular endothelial growth factor. ^a^ *p* ≤ 0.01 and ^b^ *p* ≤ 0.05 compared to baseline; all values are expressed as mean ± SD, standard deviation.

## Data Availability

The original contributions presented in this study are included in the article. Further inquiries can be directed to the corresponding author.
